# Microstructure and Selective Corrosion of Alloy 625 Obtained by Means of Laser Powder Bed Fusion

**DOI:** 10.3390/ma12111742

**Published:** 2019-05-29

**Authors:** Marina Cabrini, Sergio Lorenzi, Cristian Testa, Fabio Brevi, Sara Biamino, Paolo Fino, Diego Manfredi, Giulio Marchese, Flaviana Calignano, Tommaso Pastore

**Affiliations:** 1 Consorzio INSTM, via G. Giusti, 9, 50121 Firenze, Italy; sergio.lorenzi@unibg.it (S.L.); cristian.testa@unibg.it (C.T.); tommaso.pastore@unibg.it (T.P.); 2 University of Bergamo, Department of Engineering and Applied Sciences, Viale Marconi 5, 24044 Dalmine, Italy; 3 OMB Valves SpA, Via Europa, 7, 24069 Cenate Sotto, Italy; fabio.brevi@ombvalves.com; 4 Department of Applied Science and Technology, Politecnico di Torino, Corso Duca degli Abruzzi 24, 10129 Torino, Italy; sara.biamino@polito.it (S.B.); paolo.fino@poilito.it (P.F.); giulio.marchese@polito.it (G.M.); 5 Center for Sustainable Futures Technologies-CSFT@POLITO, Istituto Italiano di Tecnologia, Via Livorno 60, 10144 Torino, Italy; diego.manfredi@iit.it; 6 Department of Management and Production Engineering, Politecnico di Torino, Corso Duca degli Abruzzi 24, 10129 Torino, Italy; flaviana.calignano@polito.it

**Keywords:** additive manufacturing, corrosion, alloy 625, selective corrosion, oil and gas, materials qualification

## Abstract

The effect of microstructure on the susceptibility to selective corrosion of Alloy 625 produced by laser powder bed fusion (LPBF) process was investigated through intergranular corrosion tests according to ASTM G28 standard. The effect of heat treatment on selective corrosion susceptibility was also evaluated. The behavior was compared to commercial hot-worked, heat treated Grade 1 Alloy 625. The morphology of attack after boiling ferric sulfate-sulfuric acid test according to ASTM G28 standard is less penetrating for LPBF 625 alloy compared to hot-worked and heat-treated alloy both in as-built condition and after heat treatment. The different attack morphology can be ascribed to the oversaturation of the alloying elements in the nickel austenitic matrix obtained due to the very high cooling rate. On as-built specimens, a shallow selective attack of the border of the melt pools was observed, which disappeared after the heat treatment. The results confirmed similar intergranular corrosion susceptibility, but different corrosion morphologies were detected. The results are discussed in relation to the unique microstructures of LPBF manufactured alloys.

## 1. Introduction

Alloy 625 (UNS N06625) is one of the most used material in chemical and oil and gas industries for its corrosion resistance in either oxidant or non-oxidant acids. It is also used in the aerospace industry thanks to the resistance to thermal oxidation [1,2]. In the oil and gas industry, high aggressiveness of the environment requires the use of materials with outstanding corrosion resistance to ensure the reliability of assets, reduce the risk of breakage, prevent consequences on the environment and guarantee the safety of people. The continuous growth of demand and the depletion of reservoir lead the oil and natural gas extraction industry to exploit deeper wells, with extreme conditions (High Pressure High Temperature Wells—HPHT). The use of traditional alloys with low both mechanical and corrosion resistance is not suitable under such hypothesis and international standards i.e., NACE MR 0175/ISO 15156 defines such environment as “sour” even in presence of few ppm of H_2_S in the formation gas due to the high operating pressures. Materials resistant to sulfide stress corrosion cracking (SSCC) are required in these environments and high temperatures, high fraction of water in the formation and presence of CO_2_ and H_2_S implies the use of general and localized corrosion resistant alloys (Corrosion Resistance Alloys—CRA). Inconel 625^®^ (Alloy 625) is a nickel-based superalloy strengthened by solid-solution hardening of Nb and Mo in a Ni-Cr matrix [3]. Alloy 625 has a combination of high yield strength, fatigue strength, and excellent corrosion resistance in aggressive environments, it has found widespread applications in the aerospace, marine, and nuclear industries where complex shapes are often required. The alloy shows general corrosion rates less than 0.5 mm/year in concentrated non-oxidizing organic and inorganic acids [4,5]. The resistance to pitting is very high, much higher than the traditional austenitic stainless steels. Thanks to the high nickel content, it is immune to chloride stress corrosion (SCC, Stress Corrosion Cracking). Alloy 625 is precipitation-hardening alloy, due to the formation of very fine phases, which strengthen the austenitic matrix, because of aging treatments between 550–750 °C. The formation of these phases or their decomposition modifies both the yield strength and ductility of the alloy [6]. Furthermore, the formation of carbides and secondary phases can also affect the corrosion resistance. In particular, the alloy can become susceptible to intergranular corrosion if subjected to improper solubilization treatment [7].

In the last years, a very promising additive manufacturing (AM) technique has been developed in order to find alternative processes based on Laser Powder Bed Fusion (LPBF) of metal powders—layer by layer—by using a laser following the 3D CAD model. This production method has advantages in terms of cost reduction and lead time, because it eliminates swarf—typical of traditional subtractive machining—welding operations, and assembly phases. Lot of efforts has been devoted to AM of Alloy 625 alloy [8,9,10,11,12,13,14]. Alloy 625 is sensitive to precipitation of intermetallic phases such as Ni_3_M phases, Ni_2_(Cr, Mo) Laves phase as well as MC primary carbides and M_6_C and M_23_C_6_ secondary carbides. Large amount of literature exists on the heat-treatment-induced phase evolution of wrought Alloy 625 [3,15,16]. The effect of post-weld heat treatment has been also studied by several groups [17,18,19]. As AM techniques are processes characterized by melting and solidification, microstructures are expected to be different due to different thermal gradients. Precipitation of phases that occurs in AM Alloy 625 are not present at all in wrought material or they only appear after tens to hundreds of hours [2].

Despite huge amount of data and scientific papers regarding the mechanical, physical and microstructural characterization of AM of nickel-based alloys, on the authors knowledge there are no works on the corrosion behavior of the alloy obtained by means of LPBF. On the contrary several works underline the effect of the different microstructures obtained by means of LPBF on the corrosion behavior of Ti6Vl4V alloy [20,21,22], on AISI 316L [23,24,25], high strength steels [26] on Cr/Co alloys [27,28] and AlSi10Mg [29,30,31,32,33,34].

The relation between microstructure and corrosion resistance is of fundamental importance for the qualification of materials selected for hostile environments. The paper is devoted to the study of susceptibility to intergranular corrosion of Alloy 625 produced by LPBF in as-built condition and after typical heat treatment usually recommended for oil and gas service. The behavior of LPBF alloy has been compared to hot-worked, heat treated commercial Alloy 625.

## 2. Materials and Methods

**Figure 1:**
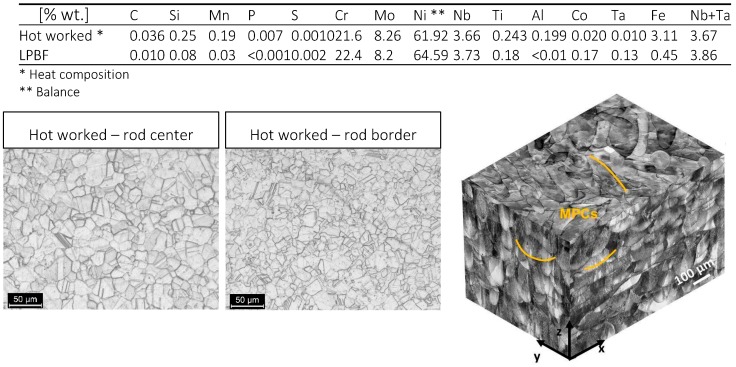
Chemical composition and 3D optical image composite of as-built IN625 sample showing the melt pool contours (MPCs) along the building direction (z-axis) and perpendicular to the building direction (x-y) plane; mixed acids reagent was used.

Commercial gas atomized Alloy 625 powder was used for LPBF process and hot-worked bar with 16 mm diameter was considered for comparison purposes. The bar was furnished in annealed condition, after heat treatment at 980 °C for 32 min and water quenching (Grade 1, according to ASTM B446 standard). The chemical composition of LPBF and hot-worked alloys as well as microstructures are shown in Figure 1. The microstructure was revealed by grinding with silicon carbides emery papers up to 4000 grit, polishing with diamond paste up to 1 μm, and then etching with Kalling’s N°2 reagent or electrolytic oxalic acid according to ASTM A262—Practice A. As-built LPBF specimens were etched by Mixed Acid Solution (15 mL HCl, 10 mL H_3_COOH and 10 mL HNO_3_, Carlo Erba RPA reagents, Cornaredo, Milan, Italy) (Figure 1). The microstructures of LPBF specimens were observed on planes parallel or perpendicular to the building direction, whereas hot-worked specimens were only examined on transverse plane.

### 2.1. Specimens

Cubic specimens of 15 mm side were printed by means of an EOSINT M270 (EOS Gmbh, Krailling, Germany) Dual Mode version working under argon atmosphere. The device operates at 195 W laser power, 1200 mm/s scan speed, 0.09 mm hatching distance as well as 0.02 mm of layer thickness. The scanning strategy involves the rotation of laser beam of 67° between consecutive layers based on the EOS strategy. The combination of the process parameters and scanning strategy allows the production of parts with a residual porosity less than 0.1%. More in-depth information about optimization procedure and building parameters were previously published [14,35]. Specimens in as-built condition and after post-processing annealing heat treatment at 980 °C for 32 min were considered. Five millimeters high cylindrical specimens were obtained by cutting the hot-worked bar.

### 2.2. Corrosion Tests

Boiling ferric sulfate/sulfuric acid corrosion tests were carried out according to ASTM G28 standard, method A. Before testing, the surface of specimens was grinded up to 1200 grit by silicon carbides emery paper. Two faces of LPBF specimens were ground and polished up to 1 μm diamond paste in order to permit observations of corrosion morphology on main planes, i.e., XY plane and XZ plane parallel and perpendicular to the building plane, respectively. One face was polished for observing morphology on plane transversal to rolling direction on disk specimens. Afterwards, the specimens were degreased in acetone in ultrasonic bath, rinsed in water and dried before their immersion in the boiling test solution. Before and after tests, the specimens were weighed by means of analytic scale to measure the weight loss and corrosion rate.

## 3. Results and Discussion

Table 1 shows the results of 120 h weight loss tests in boiling ferric sulfate/sulfuric acid solutions (ASTM G28 Method A). The highest corrosion rate was observed on hot-worked alloys. The LPBF specimens showed about the half and the third the corrosion rate measured on hot-worked specimens in as-built conditions and after heat treatment at 980 °C for 32 min, respectively. The corrosion morphology of these specimens is flat and even. On the contrary, hot-worked alloy showed penetrating corrosion attack, more severe compared to LPBF specimens.

**Figure 2:**
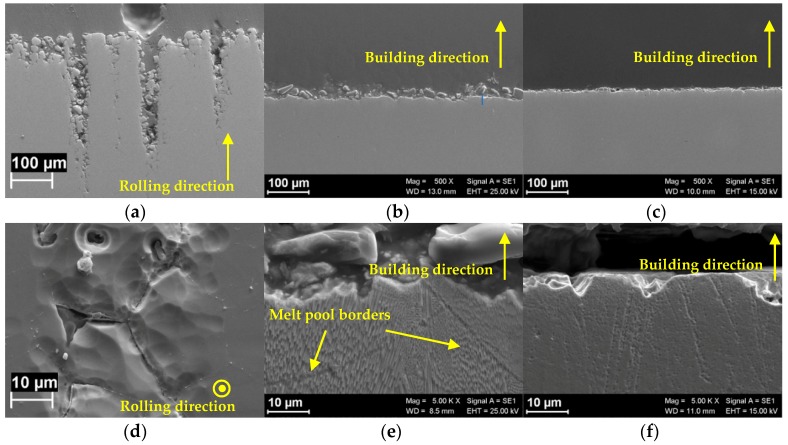
SEM images of Alloy 625 after intergranular corrosion tests. Metallographic section of commercial rolled alloy along the rolling direction (**a**) detail of the attack on the surface perpendicular to the rolling direction (**d**). Metallographic section of LPBF alloys along the building direction in as produced conditions (**b**,**c**) and after heat treatment (**e**,**f**).

**Figure 3:**
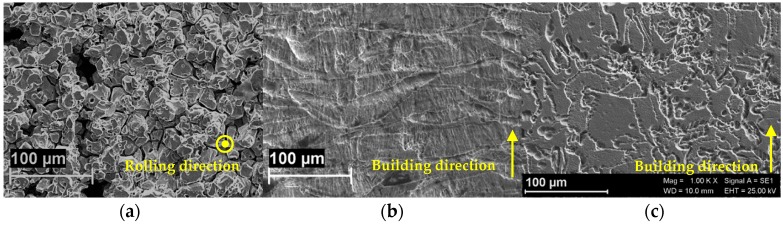
SEM images of the polished surface of the specimens after the tests of intergranular corrosion. (**a**) hot-worked bar, (**b**) LPBF as-built, (**c**) LPBF heat treated.

Figure 2 and Figure 3 show the corrosion morphology of the specimens after 120 h exposure tests in boiling ferric sulphate/sulfuric acid solution according to ASTM G28 standard. The building direction of LPBF specimens and the rolling direction of the hot-worked bar is represented by the yellow arrows. Figure 2 shows the images of the metallographic sections of the alloys taken at low and high magnification. HW specimens showed penetrating intergranular attack mainly located at the center of the specimen (Figure 2a,d).

The intergranular attack produced also grain dropping, as evidenced by the analysis of the surface after exposure (Figure 3a). The high value of the corrosion rate is practically due to grain dropping. The morphology of the corrosion attack on LPBF as-built is far more even (Figure 2b,e), but the attack follows different paths. The morphological analysis emphasizes the unique melt pool microstructure-typical of LPBF processing—formed by overlapped laser scan traces (Figure 3b). The melt pool structure is well-defined and penetrating attacks along the borders can be clearly detected. Finally, the attack takes place in form of shallow attacks on the heat treated LPBF specimens (Figure 2c,f) and the melt pool borders are no more evident. Equiaxed grain microstructure is evidenced by the corrosion attack (Figure 3c).

**Figure 4:**
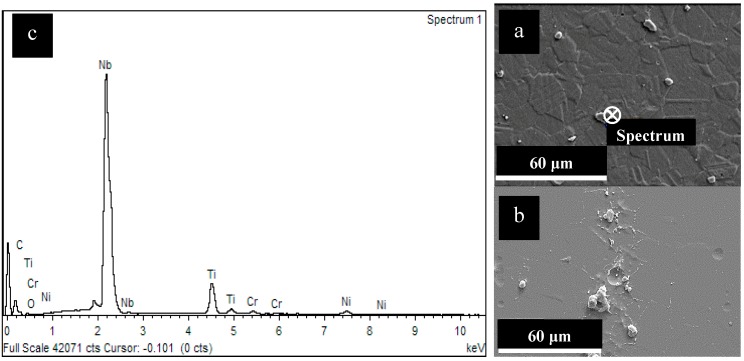
Microstructure of HW specimens (**a**,**b**) (Kalling’s attack) and EDS spectrum of macro-precipitates (**c**) taken in the point evidenced in (**a**).

The differences in the morphology of attack is strictly related to the microstructure of the alloy, which depends upon the manufacturing process and the heat treatments. HW alloy shows a deep intergranular attack very close to the axis of the bar, where strong segregation of several precipitates at the grain boundary occurred. This is well evidenced by the presence of certain amount of both macro and micro-precipitates (Figure 4). The EDS analysis carried out on these precipitates confirmed the presence of Nb-rich phases, mainly MC carbides.

Owing Kim and Lee, the formation of carbides can be a factor contributing to the degradation of corrosion resistance of Alloy 625, because intergranular areas of carbides are thermodynamically more unstable and react more readily than other intergranular ones [36]. Cortial et al. studied the effect of heat treatment on the corrosion resistance of forged and welded nickel superalloys [37,38]. They concluded that hardening between 650 and 800 °C results in dislocation gliding of matrix, γ″ precipitation and maximum of intergranular precipitation of fine M_23_C_6_ carbides. Tensile strength and intergranular corrosion become maxima, impact strength and reduction of area become minima in the field of M_23_C_6_ and M_6_C intergranular precipitation between 700 and 950 °C. Furthermore, the carbides have a higher Cr concentration than that in the base metal and they deplete the surrounding matrix in chrome, thus decreasing its corrosion resistance. However, the precipitates founded in this works were mainly enriched in Nb. The role of Nb rich precipitates in the corrosion of Alloy 625 alloy was underlined by Tawancy et al. [39]. Authors made tests in boiling 10% nitric acid on Alloy 626 annealed and aged at different time and temperature. They concluded that Ni_3_Nb phase formed during aging is preferentially corroded owing the lower chromium content compared to the surrounding matrix. The intense intergranular corrosion attack observed on the hot-worked and heat treated reference alloy can be then ascribed both to the precipitation of MC carbides and Ni_3_Nb phase at the grain boundary which is favored at the center of the bar due to the production process.

**Figure 5:**
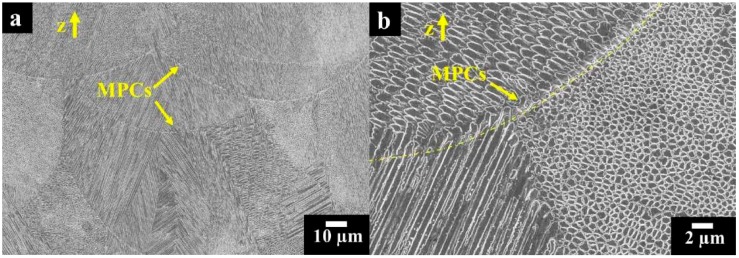
FESEM images at different magnification (**a**,**b**) exhibiting melt pools with columnar and cellular primary dendrites for as-built IN625 sample; mixed acids reagent.

The intergranular attack is not so penetrating in as-built LPBF Alloy 625 alloy, and it is not detectable at all on the LPBF alloy after the heat treatment. The microstructure of the as-built samples obtained in this work shows columnar grains (CGs) developing epitaxially, thus crossing several melt pools along the building direction (z-axis) (Figure 5). The marks underline the different melt pool borders (MPCs) along both the building direction (z-axis) and perpendicular to the building platform (x-y plane). The melt pools are not all placed straight along the building direction due to the scan strategy which involves laser beam rotation of 67° between two consecutive layers [14,40,41].

During the LPBF process, the solidification rate is different between the upper and lower zones of the melt pool. In the lower part of the layer the cooling speed is very high, while the upper part cools more slowly.

Figure 5a reveals the FESEM (Field Emission Scanning Electron Microscope) image of as-built Alloy 625 samples along the building direction. The melt pools are made up of very fine primary dendritic structures which have both cellular and columnar shapes, as shown in Figure 5b. The cellular dendrites structure is due to the alteration of columnar dendrite structures caused by very high cooling rates, as previously reported [42].

**Figure 6:**
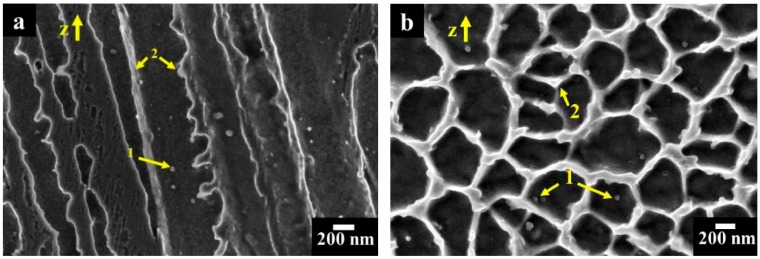
FESEM images of as-built IN625 sample at high magnification (**a**) columnar dendritic structures with nanoprecipitates (1) and segregation of Nb and Mo within the inter-dendritic zones (2); (**b**) cellular dendritic structures with nanoprecipitates (1) and segregation of Nb and Mo within the inter-dendritic areas (2).

At higher magnification (Figure 6), very fine bright precipitates along the dendritic areas indicated by arrows 1 and bright elongated phases along the inter-dendritic regions pointed out by arrows 2 can be observed. Previous TEM investigations performed on as-built Alloy 625 (produced with the same process parameters) [43] reported a relatively high concentration of Nb and C for these precipitates, suggesting the early stage formation of fine MC carbides. EDS results revealed enrichment in Nb and Mo within the inter-dendritic areas, indicated possible segregation of Nb and Mo due to their elevated tendency to segregate, as confirmed by the literature [44,45].

These precipitates did not influence the corrosion resistance of the alloys owing to their very small sizes. On the other hand, the attack seems to be preferentially located along the border of the melt pools.

Dinda et al. hypothesized the precipitation of intermetallic γ″ [Ni_3_Nb] and δ [Ni_3_Nb] phases in the heat affected zone at the border of melt pool [9], taking into account also the reticular parameters modifications in the Ni matrix. Anam et al. reported the presence of γ″ [Ni_3_Nb] precipitates at the border of melt pool [46]. A similar attack morphology was observed also for aluminum alloys [29,32,34]. However, the preferential attack at the border of the melt pool does not penetrate in depth for Alloy 625.

**Figure 7:**
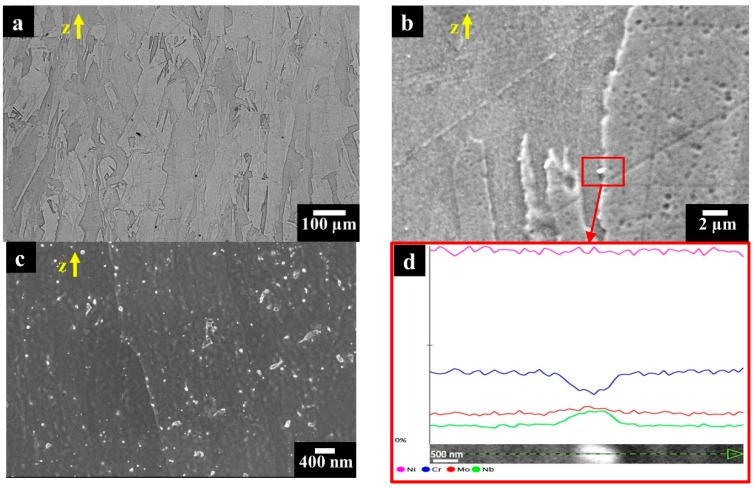
(**a**) Optical microscopy image of LPBF Alloy 625 annealed at 980 °C for 32 min and water quenched showing columnar grains along the building direction; (**b**,**c**) FESEM images of IN625 sample annealed at 980 °C for 32 min and water quenched revealing fine precipitates throughout the material, with the largest located along the grain boundaries; (**d**) EDS scan line (at higher magnification) on a carbides showing the enrichment in Nb and the depletion of Cr with respect to the austenitic matrix.

The heat treatment at 980 °C for 32 min dissolved the melt pool microstructure obtained by the LPBF process (Figure 7a). The specimens presented columnar grains along the building direction, coupled with the formation of fine carbides as displayed in Figure 7b,c. The carbides had dimensions from nanometric size (around 40 nm) as well as larger size roughly around 500 nm, as can be seen in Figure 7c,b, respectively. In order to detect a chemical composition variation, the EDS analysis was performed on the largest carbides.

EDS scan line (Figure 7d) revealed that the carbides are enriched in Nb and depleted of Cr suggesting the formation of MC carbides, which is in accordance with the TTT (Time Temperature Transformation) diagram of Alloy 625 [47].

The very low susceptibility to intergranular corrosion of the heat-treated specimens could be attributed to the absence of macroscopic precipitation as the size and distribution of the precipitate are coherent with the rounded pits on the surface of the specimen at the end of the intergranular corrosion test (Figure 7a). The precipitation of second phases is not continuous. This promotes a quite even not penetrating corrosion attack that does not penetrate significantly in depth along the grain boundaries, without any dropping. Thus, very low loss of weight was measured due to this fact.

## 4. Conclusions

The susceptibility to selective corrosion behavior of Alloy 625 obtained by means of LPBF was studied. The behavior was compared to commercial hot-worked, heat treated Grade 1 Alloy 625. The morphology of attack after boiling ferric sulfate-sulfuric acid test according to ASTM G28 standard is less penetrating for LPBF 625 alloy compared to hot-worked and heat treated alloy both in as-built condition and after heat treatment at 980 °C for 32 min. Furthermore, lower weight loss of LPBF alloy were measured.

The different attack morphology can be ascribed to the oversaturation of the alloying elements in the nickel austenitic matrix obtained due to the very high cooling rate. The precipitates of second phases along and inside the micro-dendrites are too small to promote preferential dissolution paths. On as-built specimens, a shallow selective attack of the border of the melt pools was observed. The Grade 1 heat treatment at 980 °C for 32 min followed by water quenching caused the melt pools microstructure to disappear and prevent the in-depth penetration of the selective attack.

## Figures and Tables

**Table 1:** Results of 120 h weight loss test in boiling ferric sulphate/sulfuric acid solution (ASTM G28).

Metallurgical Condition	Specimens	Exposed Surface [cm^2^]	Initial Mass * [g]	Final Mass * [g]	Mass Variation [g]	Corrosion Rate [mdd]
Hot worked bar/Grade 1 annealing	HW1	6.05	6.73923	6.63074	0.10849	359
	HW2	6.13	7.16643	7.06459	0.10184	332
LPBF as built	LPBF NHT1	13.50	28.23744	28.12392	0.11352	168.2
	LPBF NHT2	13.35	27.03907	26.92816	0.11091	166.2
LPBF/Grade 1 annealing	LPBF HT1	13.06	28.24230	28.16770	0.0746	114.2
	LPBF HT2	13.12	28.34171	28.26959	0.07212	109.9

* Average of 3 measurements.
